# Improvement in Taste Quality of Rice Porridge Using Konjac Glucomannan

**DOI:** 10.3390/foods13193146

**Published:** 2024-10-02

**Authors:** Sixuan Li, Wenhui Zhang, Min Zhang, Lina Guan, Guodong Ye

**Affiliations:** 1 School of Food and Health, Beijing Technology and Business University, 11 Fucheng Road, Beijing 100048, China; lisixuan1929@163.com (S.L.); 15010854117@163.com (W.Z.); guanlina202302@126.com (L.G.); 2350201025@st.btbu.edu.cn (G.Y.); 2 Beijing Engineering and Technology Research Center of Food Additives, Beijing 100048, China; 3 National Grain Industry Highland Barley Deep Processing Technology Innovation Center, Beijing 100048, China

**Keywords:** rice porridge, konjac glucomannan, taste quality, sensory evaluation

## Abstract

Improving the taste quality of rice porridge in a scientific way is essential to guiding residential and commercial production. This study aimed to determine the impact of konjac glucomannan (KGM) on the taste quality of rice porridge. The obtained results showed that the optimal concentration of KGM added to rice porridge is 1%. This was primarily observed via the reduction in water absorption, swelling power, and iodine blue value, thus increasing the hardness of rice porridge. At the same time, KGM also reduced the free water content and improved the water-holding capacity of rice porridge. Nevertheless, the pasting properties showed that the high viscosity of KGM raised the pasting viscosity of rice flour, thereby enhancing the stickiness of rice porridge with the increase in the KGM concentration. In addition, KGM gave the rice porridge a stronger aroma. Sensory evaluations further confirmed significant improvements in the color, odor, palatability, and overall acceptability of KGM-supplemented rice porridge (*p* < 0.05). To summarize, adding an appropriate amount of KGM was beneficial in forming a richer flavor and improving the taste quality of rice porridge. The findings offer valuable insights into the scientific processing of rice porridge products.

## 1. Introduction

Rice is one of the world’s major food crops, relying on more than 50% of the world’s population as a primary source of calories due to its high nutrient content and low micronutrients [1]. Rice products mainly include cooked rice, instant rice, rice porridge, sushi, and rice noodles. Among them, rice porridge is a rice-based liquid to semi-solid food with a soft texture, making it suitable for consumption by individuals across all age groups, especially children and the sick [2]. However, rice porridge was normally classified as a starchy food with poor taste and grain integrity and lacked functional ingredients [3]. With the progress of society, the development of a high-quality rice porridge was a good way to meet the current demand. The processing of traditional rice porridge was mainly divided into four steps: rice selection, washing, water soaking, and cooking [2,4]. Mi et al. analyzed the effect of 21 kinds of *japonica* rice from Northeast China on the flavor profiles of rice porridge [4]. Zhang et al. found that soaking conditions, pH value, cooking temperature, and pressure could affect the taste quality of rice porridge [5]. Generally, various factors, including rice varieties, cooking techniques, and additives, all contribute to the palatability of rice porridge. Non-starch polysaccharides, as high-quality additives, affected the pasting and retrogradation properties of starch and could be applied to starch-based foods to modify the texture and control water migration, thus improving the taste quality. Konjac glucomannan (KGM) is a kind of natural water-soluble and neutral non-starch polysaccharide extracted from konjac tuber, which has been widely used in the food industry [6]. Ren et al. mixed glutinous rice flour with KGM to make mochi, which improved the texture and sensory quality of mochi [7]. Lu et al. found that KGM improved the freeze–thaw stability of dough [8]. Nagasawa et al. found that adding KGM to rice porridge could control the increase in plasma glucose and insulin levels after meals [9]. However, few studies have investigated the effect of KGM on the palatability and flavor of rice porridge.

Therefore, the present study was carried out to investigate the impact of different concentrations of KGM on the cooking and pasting properties of rice, as well as its effect on the color, texture, flavor, water distribution, and sensory qualities of rice porridge. This study aimed to develop a rice porridge product with a palatable texture and pleasant flavor by adding KGM. Also, it was a great option to improve the acceptance and enhance the sensory qualities of rice porridge, which helped to produce rice porridge that was healthier and more sustainable. Furthermore, the findings might provide a theoretical foundation for the high-value utilization of KGM.

## 2. Materials and Methods

### 2.1. Materials

Liaojing 433 (*Japonica* rice, protein: 6.26%, lipid: 1.33%, amylose: 16.40%, amylopectin: 52.80%, moisture: 13.35%) was provided by Liaoning Agricultural Science and Technology Co., Ltd. (Shenyang, China). They were harvested in 2023 and stored at −20℃. Before milling, the rice was placed at room temperature for 30 min and then hulled and milled (30 s for hulling and 80 s for milling). KGM (food grade, purity: 95%) was purchased from Hubei Konson Konjac Technology Co., Ltd. (Ezhou, China). Other reagents were purchased from Jinlis Co., Ltd. (Beijing, China).

### 2.2. Preparation of Rice Porridge

Based on the results of the previous study on the concentration gradient of KGM, a certain mass of KGM was weighed and prepared into solutions of different concentrations based on the mass of rice, and the procedure was slightly modified according to the method of Jowee and Brownlee [10]. Ten grams of rice were soaked in purified water at a ratio of 1:7 for 30 min and finally steamed on an electric steamer for 30 min. The control group and rice porridge with 0.5%, 1.0%, 1.5%, and 2% of KGM (*w*/*w*, based on rice mass) were named CON, KGM-0.5, KGM-1.0, KGM-1.5, and KGM-2.0, respectively.

### 2.3. Boiling Properties of Rice Grain

We followed the method of Wang et al. with slight modifications [11]. Seven grams of rice grains were weighed and placed in a 100 mL measuring cylinder, 25 mL of KGM solution was added, the volume (V_1_) was read and transferred to a beaker (M_1_), 120 mL of KGM solution was added, and the mixture was steamed using an electric steamer for 20 min. Then, the rice soup was drained and transferred to a cone-shaped bottle, the weight of the beaker and rice (M_2_) were weighed after cooling, the rice was transferred to a 100 mL measuring cylinder, and 50 mL of KGM solution was added to read the volume (V_2_). The water absorption and swelling power were calculated according to Formulas (1) and (2), respectively.

(1) $$Water\;absorption\;(\%)\;=\frac{M_{2}-M_{1}}{7.0}\times 100\%$$

(2) $$Swelling\;power\;(\%)\;=\frac{V_{2}-50}{V_{1}-25}\times 100\%$$

After cooling, the rice soup was diluted into a volumetric bottle, and then 30 mL was added to a centrifuge tube. The insoluble solid impurities were removed by centrifugation at 4000 rpm for 15 min. Subsequently, 10 mL of the supernatant was drawn into a pre-dried aluminum dish (W_1_), dried to a constant weight at 110 °C in an oven, cooled, and weighed (W_2_). The dry matter was calculated according to Formula (3):

(3) $$Dry\;matter\;(mg/g)=\frac{W_{2}-W_{1}}{7.0}\times \frac{100}{10}$$

Then, 0.5 mol/L of HCl (5 mL) and 0.2 g/100 mL of iodine reagent (1 mL) were added to the supernatant, made up to 25 mL. After standing for 10 min, the absorbance value was determined at a wavelength of 660 nm by a spectrophotometer (UV-2800A, Unico Instrument Co., Ltd., Shanghai, China), i.e., the iodine blue value was obtained.

### 2.4. Pasting Properties of Rice Flour

The pasting properties of rice flour were assessed utilizing the method described by Jia et al. [12]. Three grams of rice flour were mixed with 25 g of various concentrations of the KGM solution. The test procedure was heated to 50 °C for 1 min, followed by an increase to 95 °C at 12 °C/min for 2.5 min, and then a decrease to 50 °C at the same rate. The first 10 s of rotating stirring speed was 960 rpm, followed by 160 rpm until the end.

### 2.5. Color of Rice Porridge

We used a colorimeter (CR-400, Konica Minolta, Tokyo, Japan) to analyze the appearance quality of rice porridge, calibrated with a white tile (Y = 81.9, x = 0.3222, c = 0.3401) before testing. Subsequently, the L*, a*, and b* values of rice porridge were measured, and then the whiteness (W) was calculated based on Formula (4) [13]:

(4) $$W=100-\sqrt{{\left(100-L^{*}\right)}^{2}+{a^{*}}^{2}+{b^{*}}^{2}}$$

where W is the whiteness, L* is the brightness, a* is the red-green value, and b* is the yellow-blue value.

### 2.6. Water Mobility and Distribution of Rice Porridge

The rice porridge (1.5 g) was weighed in a liquid phase vial and placed in an NMR tube [14]. The resonance center frequency was adjusted using the FID signal. Subsequently, the Carr–Purcell–Meiboom–Gill (CPMG) sequence was used to determine the transverse relaxation time (*T*_2_). The test parameters were as follows: P2 = 35.04 μs, P1 = 17.00 μs, TW = 5500 ms, TD = 150,042, NECH = 1500, TE = 0.5 ms, and NS = 32.

### 2.7. Texture Properties of Rice Porridge

According to our previous method, three whole rice grains were randomly selected from the rice porridge and arranged in a radiative shape [14]. The texture analyzer was set to the TPA test mode and utilized the P/36R probe, with the following parameter settings: trigger force of 0.05 N, test speed of 30 mm/min, and compression ratio of 70%.

### 2.8. Flavor Profiles of Rice Porridge

#### 2.8.1. Electronic Nose (E-Nose)

The E-nose was used to distinguish the aromas in different rice porridge samples [15]. The responsive substance of the different sensors is described in Table S1. Rice porridge (5.0 g) was weighed into a headspace vial and allowed to equilibrate for 10 min before measurement. The sensor parameters were set as follows: an injection flow rate of 300 mL/min, a cleaning time of 100 s, and an analysis time of 60 s.

#### 2.8.2. Gas Chromatograph-Mass Spectrometer (GC-MS)

Solid-phase microextraction was used to extract the volatile compounds from rice porridge [14]. Six grams of rice porridge were weighed in a headspace vial, and then 2-methyl-3-heptanone (1 μL, 0.816 μg/μL) was added. The adsorptive extraction was carried out for 40 min after incubation for 15 min in a stirrer at 80 °C, followed by GC-MS analysis. The parameters were set as follows: the flow rate of helium was 1.2 mL/min, and the initial temperature was 40 °C and held for 3 min, then increased to 200 °C at 5 °C/min, and then increased to 230 °C at 10 °C/min. The content of volatile compounds was calculated based on Formula (5):

(5) $$C(\mu g/kg)=\frac{P_{C}}{P_{is}}\times C_{is}\times 1000/m_{0}$$

where *C* is the content of volatile compounds, $C_{is}$ is the content of 2-methyl-3-heptanone, $P_{c}$ is the peak area of volatile compounds, $P_{is}$ is the peak area of 2-methyl-3-heptanone, and $m_{0}$ is the weight of rice porridge.

### 2.9. Sensory Evaluation of Rice Porridge

A total of 14 evaluators (8 females, and 6 males, aged 21–30) were selected and trained in sensory evaluation. This study was approved by the Ethics Committee of Beijing University of Business and Economics (Ethics Approval 2024 No. (161)), and informed consent was obtained from each participant before testing. The evaluation was conducted based on the scoring guidelines developed in our previous studies (Table S2) [4].

### 2.10. Statistical Analysis

All experiments were replicated at least three times, and the data were expressed as “mean ± standard deviation”. One-way analysis of variance (ANOVA) and Duncan’s multiple comparison tests were used to identify differences between samples, with *p* < 0.05 considered statistically significant. ChiPlot (https://www.chiplot.online/, accessed on 2 May 2024) and Origin 2023b (Origin Lab Corp., Northampton, MA, USA) were utilized for mapping.

## 3. Results and Discussion

### 3.1. Boiling Properties Analysis

The boiling properties of rice grain with KGM added are presented in Table 1. Both water absorption and swelling power exhibited dose-dependent phenomena in the KGM-added rice porridge samples. As the concentration of KGM increased, the water absorption and swelling power of rice grain showed a decreasing trend. This might be attributed to the fact that KGM competed with rice starch for water molecules due to its strong water-holding capacity, thus limiting the formation of hydrogen bonds between starch and water molecules [16]. The iodine blue value reflected the amount of amylose dissolved in water. Adding KGM had been found to reduce the leached amylose, consequently leading to a significant decrease in the iodine blue value [17]. When the concentration of KGM increased to 1.5%, the iodine blue value no longer changed significantly. This might be due to the fact that the inhibition effect on starch gelatinization at KGM concentrations of 1.5% and 2.0% were similar. Changes in the boiling properties of rice grain directly affected the taste quality of rice porridge, similar to a recent study [18].

### 3.2. Pasting Properties Analysis

**Figure 1:**
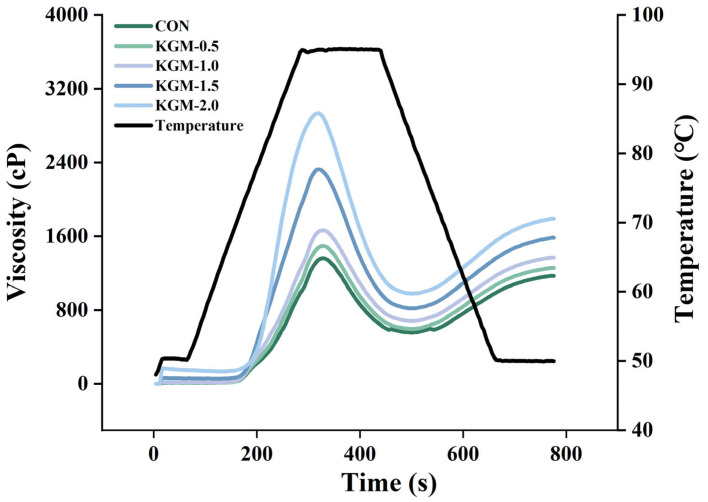
Effect of konjac glucomannan on pasting properties of rice (CON: control group, KGM-0.5, KGM-1.0, KGM-1.5, and KGM-2.0 indicated that different additions of konjac glucomannan were added to rice porridge).

The impacts of KGM on the pasting properties of rice flours are illustrated in Figure 1 and Table S3. All viscosity values increased in correlation with the increase in the KGM concentration, and the pasting temperature also showed a corresponding gradual increase. This involved two main reasons: (a) KGM had a good water absorption capacity, effectively reducing the amount of water that could be absorbed by the rice grains during heating and swelling, and further pasting could only be accomplished by increasing the temperature; and (b) KGM was entangled on the surface of rice grains, which interfered with the water absorption of starch granules [19]. Despite the inhibition of starch gelatinization, the viscosity values were still increased, which could be attributed to the thickening effect of KGM [20]. Compared to the control group, the setback value increased significantly with an increasing KGM concentration, indicating that the presence of KGM caused a more pronounced tendency of aggregation. This might be caused by the cross-linking between KGM and starch molecules [21]. At the same time, the strong water-binding capacity of KGM resulted in the more effective dispersion of starch in the continuous phase, making it easier to rearrange between starch molecules, thus resulting in a higher setback value [22].

### 3.3. Color Analysis

**Figure 2:**
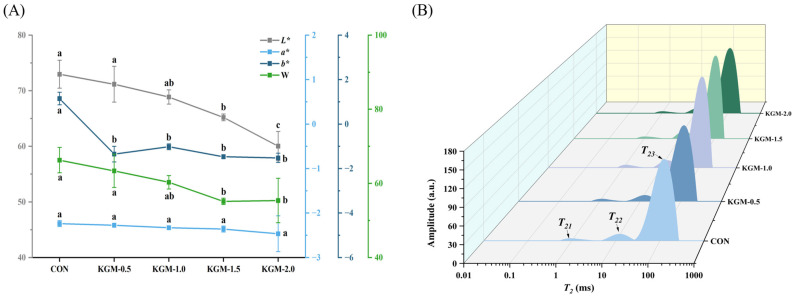
Effect of konjac glucomannan on the appearance quality (**A**) and water distribution (**B**) of rice porridge (CON: control group, KGM-0.5, KGM-1.0, KGM-1.5, and KGM-2.0 indicated that different additions of konjac glucomannan were added to rice; different letters were considered to be significantly different, *p* < 0.05).

The appearance quality of rice porridge is shown in Figure 2A. The L* value ranged from 60.04 to 72.96, and there was no significant difference between the control group and samples with KGM concentrations of 0.5% and 1.0%. As the KGM concentration increased, the L* value gradually decreased. The W value ranged from 55.37 to 66.27, showing the same trend as the L* value, indicating that the addition of KGM darkened the color of rice porridge. This was mainly because the L* and W values of KGM were lower than those of rice. KGM might also inhibit starch gelatinization, resulting in lower transparency of rice grains. In addition, adding KGM would increase the degree of the Maillard reaction during the rice porridge-making process, thus intensifying the browning reaction and making yellowish rice grains [23]. All samples had a negative a* value indicating a greenish color and there was no significant change in the a* value. The b* value was also not significantly different among the samples. Therefore, the concentration of KGM should be controlled to below 1.0% when making rice porridge.

### 3.4. Water Distribution Analysis

As shown in Figure 2B and Table S4, the transverse relaxation time (*T*_2_) reflected the freedom of water, which included bound water (*T*_21_), immobile water (*T*_22_), and free water (*T*_23_), with the corresponding peak area proportions denoted as *A*_21_, *A*_22_, and *A*_23_, respectively [12]. After adding KGM, the *T*_21_ value remained largely unchanged, whereas the *T*_23_ value showed a decreasing trend followed by an increase, indicating that KGM had a strong water-binding ability, reducing the mobility of water molecules. Consequently, the bound water within the rice grains was relatively stable [24]. However, the excessive KGM might combine with leached amylose and amylopectin molecules, thereby weakening the interaction between starch and water molecules [25]. Similarly, *A*_21_ and *A*_22_ values increased initially and then decreased, while the *A*_23_ value decreased initially and then increased. The results indicated that KGM could bind a large amount of water molecules through hydrogen bonding, enhancing the water retention of rice porridge. When the concentration of KGM increased to 2%, the *A*_21_ and *A*_22_ values were lower, indicating that the excessive KGM prevented water molecules from entering the rice grains and increased the hardness of the rice porridge. In summary, adding KGM to rice porridge would change the migration and distribution of water molecules, thereby affecting the texture properties of rice porridge.

### 3.5. Texture Analysis

**Figure 3:**
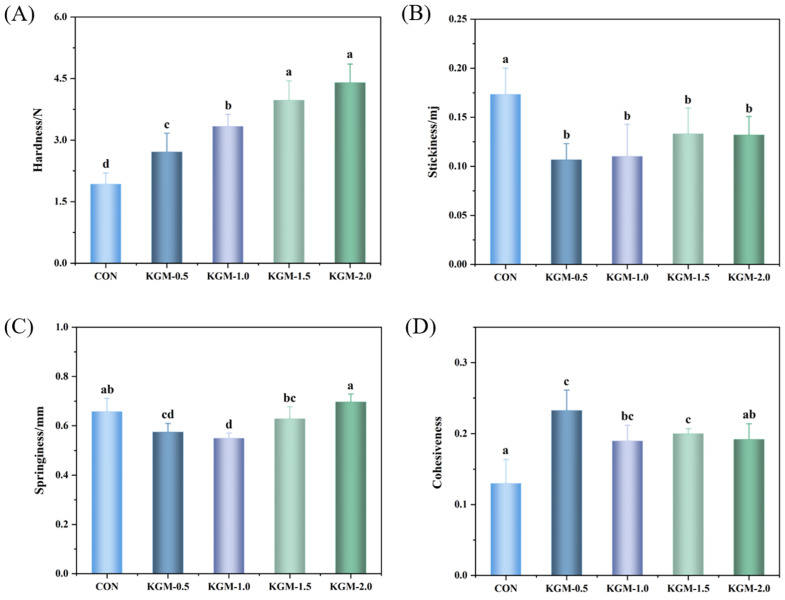
Effect of konjac glucomannan on texture properties of rice porridge. (**A**) Hardness; (**B**) stickiness; (**C**) springiness; (**D**) cohesiveness (CON: control group, KGM-0.5, KGM-1.0, KGM-1.5, and KGM-2.0 indicated that different additions of konjac glucomannan were added to rice porridge; different letters were considered to be significantly different, *p* < 0.05).

Hardness, stickiness, springiness, and cohesiveness contribute greatly to the texture of rice porridge, as shown in Figure 3A–D. The hardness of rice porridge increased gradually with the increase in the KGM concentration. However, there was no significant change in hardness when the concentration reached 1.5% and 2.0%, indicating that KGM limited the water absorption of rice, thus inhibiting the gelatinization process and increasing the hardness of rice porridge [26]. The cohesiveness also showed an increasing trend, the higher the cohesiveness within the grains, the denser the structure. Rice porridge with moderate cohesion offered a more desirable taste. Compared with the control group, the stickiness of rice porridge decreased, demonstrating that the binding ability of KGM to water molecules was significantly higher than that of starch to water molecules. Consequently, the swelling of rice grains was hindered, allowing more amylose molecules to remain in the granule without entering the continuous phase, resulting in a decrease in stickiness [27]. However, the stickiness gradually increased when the concentration of KGM increased to 1.0% and 2%, indicating that the high viscosity of KGM itself played a leading role. Figure 3C also showed that the springiness tended to decrease and then increase with an increasing KGM concentration, likely due to the combined effect of the surface damage of the rice grains and the amount of leached materials [28]. Therefore, a KGM concentration of 1.0% gave the rice porridge a pleasing texture.

### 3.6. E-Nose Analysis

**Figure 4:**
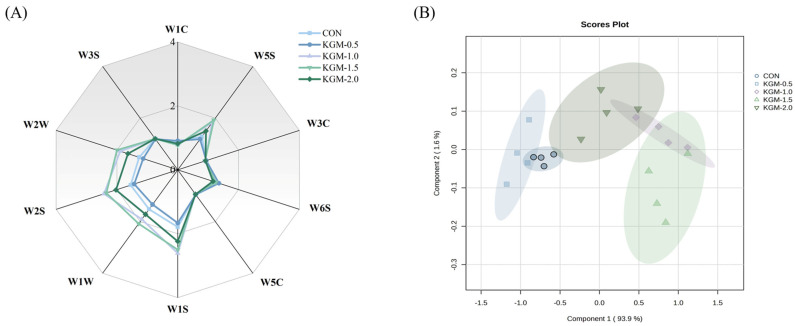
Effect of konjac glucomannan on the flavor profiles of rice porridge. (**A**) Electronic nose results; (**B**) PCA results (CON: control group, KGM-0.5, KGM-1.0, KGM-1.5, and KGM-2.0 indicated that different additions of konjac glucomannan were added to rice porridge).

The E-nose technique was employed to determine the changes in the flavor profiles of rice porridge with different concentrations of KGM, as illustrated in Figure 4. Our observations revealed distinctions in the flavor profiles among the samples, suggesting that the diverse KGM concentrations interacted with starch, protein, and fat molecules throughout the cooking process, thereby affecting the aroma release [29]. After adding KGM, the response values of W5S, W2W, W2S, W1W, and W1S sensors increased, signifying an increase in sulfides, aldehydes, and methyl compounds. The increase in these compounds could be attributed to lipid oxidation and Maillard reaction.

The principal component analysis (PCA) showed (Figure 4B) that PC1 accounted for 93.9% of the total variance and PC2 accounted for 1.6% of the total variance, suggesting that the model effectively reflected the overall flavor profiles of rice porridge. The rice porridge samples were distributed in different regions, and there was some overlap between CON and KGM-0.5 samples, but almost no overlap with other groups of samples, indicating that a certain concentration of KGM had a significant effect on the aroma compounds of rice porridge. There was an overlap between KGM-1.0 and KGM-1.5 and KGM-2.0 samples, suggesting that these three samples had similar aroma compounds.

### 3.7. GC-MS Analysis

**Figure 5:**
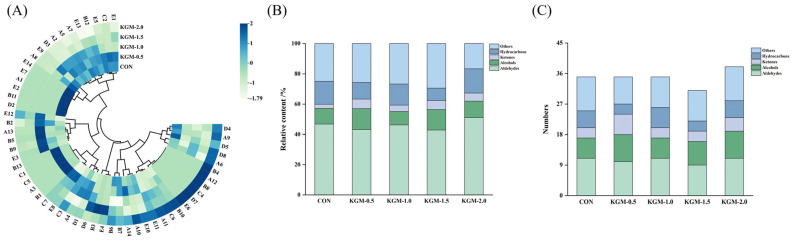
The relative content and numbers of volatile compounds in rice porridge Effect of konjac glucomannan on volatile compounds in rice porridge. (**A**) The heat map of volatile compounds; (**B**) the relative content of volatile compounds; (**C**) The numbers of volatile compounds (CON: control group, KGM—0.5, KGM—1.0, KGM—1.5, and KGM—2.0 indicated that different additions of konjac glucomannan were added to rice porridge).

As shown in Figure 5 and Table S5, a total of 56 volatile compounds were detected in all samples, including 14 aldehydes, 13 alcohols, 7 ketones, 8 hydrocarbons, and 14 heterocyclics. Aldehydes are one of the important products of lipid oxidation, mainly produced by the oxidation of polyunsaturated fatty acids. For instance, hexanal exhibits fruit-like aromas at lower concentrations, while nonanal can impart grassy and barbecue aromas to food. However, an excessive concentration of aldehydes can produce off flavors associated with oil oxidation and rancidity [30]. The total aldehyde contents in the CON, KGM-0.5, KGM-1.0, KGM-1.5, and KGM-2.0 samples were 21.22 μg/kg, 19.16 μg/kg, 20.62 μg/kg, 11.62, and 24.16 μg/kg, respectively. The aldehydes presented in all samples were hexanal, octanal, (E)-2-heptenal, nonanal, (E)-2-octenal, benzaldehyde, decanal, and (E)-2-nonenal. As the KGM concentration increased, a downward trend was observed in the content of most aldehyde compounds. Ketones, derived from lipid oxidation and the Maillard reaction, significantly influence the flavor of rice porridge [31]. Notably, 2-Pentadecanone was not detected in the control group. The two ketones, 6,10-dimethyl-6-methyl-5-hepten-2-one and (Z)-5,9-undecadien-2-one, increased with increasing KGM concentration. Also, as seen in Figure 5B, the highest content of ketones was found in KGM-0.5, suggesting that KGM promoted the formation of ketones in rice porridge. Alcohols produced secondarily from unsaturated fatty acid oxidation were the primary components in rice porridge after aldehydes [32]. The content of 2-nonen-1-ol, 1-dodecanol, and 1-octen-3-ol were significantly higher in KGM-0.5 and KGM-1.0 than in the control group, which played a vital role in enhancing the flavor of the rice porridge. Heterocyclic compounds such as 2-pentylfuran and 2, 3-dihydrobenzofuran were formed by the Maillard reaction. Their concentrations exhibited a declining trend as the KGM concentration increased, indicating that the gelatinization degree of rice starch affected the rate of Maillard reaction. In general, both KGM-0.5 and KGM-1.0 had a better flavor profile.

### 3.8. Sensory Analysis

The sensory evaluation was conducted on rice porridge with different concentrations of KGM, with the results presented in Table 2. As the concentration of KGM increased, the odor scores of rice porridge increased first and then decreased because KGM could participate in the Maillard reaction and improve the aroma of rice porridge. However, an excessively high concentration of KGM might encapsulate the aroma molecules, thus affecting their release and consequently reducing the odor scores. Similarly, the color scores of rice porridge followed a trend of an initial increase followed by a decrease, reaching the maximum at a KGM concentration of 1.0%. The subsequent decrease in color scores was likely due to the addition of KGM inhibited the starch pasting, resulting in a darker color of rice porridge [33]. For the palatability scores of rice porridge, we also observed a trend of first increasing and then decreasing. The taste scores of rice porridge in the KGM-0.5, KGM-1.0, and KGM-1.5 groups were higher than those of the control group. Consequently, the rice porridge had the highest scores in odor, appearance, and palatability when the KGM concentration was 1.0%, indicating that adding an appropriate concentration of KGM could form a richer aroma in the rice porridge and improve its overall taste quality.

## 4. Conclusions

In this study, KGM exhibited a significant effect on the taste quality of rice porridge, with the optimal effect observed at a concentration of 1.0% (*w*/*w*, based on rice mass). The appropriate concentration of KGM gave rice porridge an excellent texture, mainly due to KGM’s strong water-binding ability to compete with starch for water molecules, thus resulting in lower water absorption and swelling of rice grain, as well as a higher pasting temperature. At the same time, the pasting properties showed that the viscosity values of rice flour with KGM increased due to the high viscosity of KGM, resulting in a better viscoelasticity of rice porridge. Adding KGM also endowed rice porridge with richer flavors, reflected in the increase in ketones and alcohols that were key aromatic compounds. Sensory evaluation scores further confirmed that an appropriate concentration of KGM could improve the taste quality of rice porridge.

## Figures and Tables

**Table 1:** Effect of konjac glucomannan on the boiling properties of rice.

	Water Absorption/%	Swelling Power %	Dry Matter/%	Iodine Blue Value
CON	317.23 ± 34.30 ^a^	455.30 ± 63.21 ^a^	2.86 ± 0.18 ^a^	1.10 ± 0.09 ^a^
KGM-0.5	310.59 ± 35.71 ^a^	452.93 ± 14.58 ^ab^	2.69 ± 0.25 ^a^	0.85 ± 0.06 ^b^
KGM-1.0	289.77 ± 9.40 ^ab^	409.07 ± 6.76 ^ab^	2.55 ± 0.39 ^a^	0.59 ± 0.01 ^c^
KGM-1.5	266.53 ± 6.75 ^ab^	395.08 ± 18.00 ^ab^	2.57 ± 0.47 ^a^	0.53 ± 0.01 ^d^
KGM-2.0	235.63 ± 9.31 ^b^	366.55 ± 25.55 ^b^	2.45 ± 0.15 ^a^	0.50 ± 0.01 ^d^

CON: control group, KGM-0.5, KGM-1.0, KGM-1.5, and KGM-2.0 indicated that different additions of konjac glucomannan were added to rice. Different letters were considered to be significantly different, *p* < 0.05.

**Table 2:** Effect of konjac glucomannan on the sensory scores of rice porridge.

	Odor Scores	Appearance Scores	Palatability Scores	Taste Scores	Total Scores
CON	16.36 ± 2.41 ^ab^	18.43 ± 2.87 ^a^	22.79 ± 3.17 ^a^	20.93 ± 1.90 ^a^	78.50 ± 7.08 ^ab^
KGM-0.5	17.14 ± 1.79 ^a^	19.29 ± 2.89 ^a^	22.50 ± 1.87 ^a^	22.36 ± 1.60 ^a^	81.29 ± 5.04 ^ab^
KGM-1.0	17.29 ± 1.82 ^a^	20.50 ± 2.93 ^a^	23.93 ± 2.27 ^a^	21.93 ± 2.09 ^a^	83.64 ± 7.66 ^a^
KGM-1.5	15.71 ± 2.13 ^ab^	19.36 ± 2.79 ^a^	22.50 ± 2.85 ^a^	21.43 ± 2.28 ^a^	79.00 ± 7.24 ^ab^
KGM-2.0	15.00 ± 3.59 ^b^	18.79 ± 3.40 ^a^	22.36 ± 3.56 ^a^	20.57 ± 3.41 ^a^	76.71 ± 10.67 ^b^

CON: control group, KGM-0.5, KGM-1.0, KGM-1.5, and KGM-2.0 indicated that different additions of konjac glucomannan were added to rice. Different letters were considered to be significantly different, *p* < 0.05.

## Data Availability

The original contributions presented in the study are included in the article and Supplementary Materials, and further inquiries can be directed to the corresponding author.

## Supplementary Materials

The following supporting information can be downloaded at: https://www.mdpi.com/article/10.3390/foods13193146/s1, Table S1: The sensor array of the E-nose; Table S2: Sensory evaluation of rice porridge; Table S3: Effect of konjac glucomannan on the pasting properties of rice flour; Table S4: The proportion of different water state; Table S5: Effect of konjac glucomannan on volatile compounds of rice porridge.

## References

[1] Asimi S., Ren X., Zhang M., Li S., Guan L., Wang Z., Liang S., Wang Z. (2022). Fingerprinting of volatile organic compounds for the geographical discrimination of rice samples from Northeast China. Foods.

[2] Wang S., Tian A., Zhao K., Zhang R., Lei Z., Qin X., Wu X., Liu Y., Liu P., Yang S. (2023). Effect of cooking methods on volatile compounds and texture properties in rice porridge. LWT-Food Sci. Technol..

[3] Mayachiew P., Charunuch C., Devahastin S. (2015). Physicochemical and thermal properties of extruded instant functional rice porridge powder as affected by the addition of soybean or mung bean. J. Food Sci..

[4] Mi Y., Wang Z., Guan L., Zhang M., Li S., Ye G., Ren X., Liang S. (2023). Analysis of volatile compounds in rice porridge of different japonica rice varieties in Northeast China. J. Cereal Sci..

[5] Zhang Y., Yang N., Fray R.G., Fisk I., Liu C., Li H., Han Y. (2018). Characterization of volatile aroma compounds after in-vial cooking of foxtail millet porridge with gas chromatography-mass spectrometry. J. Cereal Sci..

[6] Ma S., Zhu P., Wang M. (2019). Effects of konjac glucomannan on pasting and rheological properties of corn starch. Food Hydrocoll..

[7] Ren B., Xie H., Guo L., Zhong K., Gao H. (2020). Effect of konjac glucomannan on sensory, physical and thermal properties of mochi. Int. J. Food Eng..

[8] Lu P., Guo J., Fan J., Wang P., Yan X. (2023). Combined effect of konjac glucomannan addition and ultrasound treatment on the physical and physicochemical properties of frozen dough. Food Chem..

[9] Nagasawa T., Kimura T., Yoshida A., Tsunekawa K., Araki O., Ushiki K., Ishigaki H., Shoho Y., Suda I., Hiramoto S. (2021). Konjac glucomannan attenuated triglyceride metabolism during rice gruel tolerance test. Nutrients.

[10] Jowee N., Brownlee I.A. (2017). The effect of high β-glucan flour incorporation into instant rice porridge on satiety and energy intake. Bioact. Carbohydr. Diet. Fibre.

[11] Wang G., Yan X., Wang B., Hu X., Chen X., Ding W. (2022). Effects of milling methods on the properties of rice flour and steamed rice cakes. LWT-Food Sci. Technol..

[12] Jia Z., Luo Y., Barba F.J., Wu Y., Ding W., Xiao S., Lyu Q., Wang X., Fu Y. (2022). Effect of β-cyclodextrins on the physical properties and anti-staling mechanisms of corn starch gels during storage. Carbohydr. Polym..

[13] Shen M., Huang K., Guan X., Xia J., Sun Z., Yu Z., Fang Y. (2023). Effects of milling on texture and in vitro starch digestibility of oat rice. Food Chem. X.

[14] Li S., Zhang M., Ren X., Guan L., Ye G., Li Y. (2024). Improvement in storage stability of fresh instant rice using non-starch polysaccharides. Int. J. Food Sci. Tech..

[15] Zhong Y., He F., Wang M., Zhang Y., Lan H., Chen L., Zeng Z. (2023). Effects of stabilization combined with fermentation treatments on the volatile composition and flavor profile of cooked black rice. Food Biosci..

[16] Nguyen T.T.L., Flanagan B.M., Tao K.Y., Ni D.D., Gidley M.J., Fox G.P., Gilbert R.G. (2021). Effect of processing on the solubility and molecular size of oat β-glucan and consequences for starch digestibility of oat-fortified noodles. Food Chem..

[17] Li K., Bao J., Corke H., Sun M. (2017). Genotypic diversity and environmental stability of starch physicochemical properties in the USDA rice mini-core collection. Food Chem..

[18] Chen H., Chen D., He L., Wang T., Ren W. (2021). Correlation of taste values with chemical compositions and Rapid Visco Analyser profiles of 36 indica rice (*Oryza sativa* L.) varieties. Food Chem..

[19] Su H., Tu J., Zheng M., Deng K., Lu X. (2020). Effects of oligosaccharides on particle structure, pasting and thermal properties of wheat starch granules under different freezing temperatures. Food Chem..

[20] Hao M., Zhu X., Ji X., Shi M., Yan Y. (2024). Effect of konjac glucomannan on structure, physicochemical properties, and in vitro digestibility of yam starch during Extrusion. Foods.

[21] Li J., Zhu M., Gu L., Su Y., Yang Y., Chang C., Han Q. (2023). Freeze-thaw stability of konjac glucomannan hydrogels supplemented with natural tapioca/corn starch. LWT-Food Sci. Technol..

[22] Zhou Y., Charles G., Yun W., Liang J., Foster T.J., Cheng Y. (2014). Effect of a small amount of sodium carbonate on konjac glucomannan-induced changes in thermal behavior of wheat starch. Carbohydr. Polym..

[23] Liu Q., Kong Q., Li X., Lin J., Chen H., Bao Q., Yuan Y. (2020). Effect of mild-parboiling treatment on the structure, colour, pasting properties and rheology properties of germinated brown rice. LWT-Food Sci. Technol..

[24] Chen R., Williams P.A., Shu J., Luo S., Chen J., Liu C. (2022). Pectin adsorption onto and penetration into starch granules and the effect on the gelatinization process and rheological properties. Food Hydrocoll..

[25] Fu Y., Liu X., Xie Q., Chen L., Wang X. (2021). Effects of Laminaria japonica polysaccharides on the texture, retrogradation, and structure performances in frozen dough bread. LWT- Food Sci. Technol..

[26] Huang M., Kennedy J.F., Li B., Xu X., Xie B.J. (2007). Characters of rice starch gel modifed by gellan, carrageenan, and glucomannan: A texture profile analysis study. Carbohydr. Polym..

[27] Zheng J., Wang N., Yang J., You Y., Zhang F., Kan J., Wu L. (2024). New insights into the interaction between bamboo shoot polysaccharides and lotus root starch during gelatinization, retrogradation, and digestion of starch. Int. J. Biol. Macromol..

[28] Zhang Y., Li F., Huang K., Li S., Cao H., Xie J., Guan X. (2023). Structural changes of starch under different milling degrees affect the cooking and textural properties of rice. Food Chem. X..

[29] Ma R., Jin Z., Wang F., Tian Y. (2021). Contribution of starch to the flavor of rice-based instant foods. Crit. Rev. Food Sci. Nutr..

[30] Ma R., Tian Y., Chen L., Jin Z. (2020). Impact of cooling rates on the flavor of cooked rice during storage. Food Biosci..

[31] Choi S., Seo H., Lee K., Lee S., Lee J., Lee J. (2019). Effect of milling and long-term storage on volatiles of black rice (*Oryza sativa* L.) determined by headspace solid-phase microextraction with gas chromatography-mass spectrometry. Food Chem..

[32] Xu J., Liu K., Zhang C. (2021). Electronic nose for volatile organic compounds analysis in rice aging. Trends Food Sci. Technol..

[33] Ding C., Khir R., Pan Z., Wood D.F., Tu K., El-Mashad H., Berrios J. (2016). Improvement in storage stability of infrared-dried rough rice. Food Bioprocess Technol..

