# Supplementary material for: Improvement in Taste Quality of Rice Porridge Using Konjac Glucomannan
Source: Foods. 2024 Oct 2;13(19):3146. doi: 10.3390/foods13193146 (PMC11475956; doi:10.3390/foods13193146)
Supplement: Supplementary file 1 [file foods-13-03146-s001.zip › foods-3213871-supplementary.pdf]

Supplementary Material

(Table S1-S5)

Sixuan Li[123], Wenhui Zhang[123], Min Zhang[123]*, Lina Guan[123], Guodong Ye[123]

1. Beijing Technology and Business University, School of Food and Health, Beijing, 100048, China

2. Beijing Engineering and Technology Research Center of Food Additives, Beijing, 100048, China

3. National Grain Industry Highland Barley Deep Processing Technology Innovation Center, Beijing, 100048, China

Address: Beijing Technology and Business University, 11 Fucheng Road, 100048, Beijing, China.

*Corresponding author: Min Zhang (zmin@th.btbu.edu.cn)

Tel: 86-10-68984547

Tables

Table S1 The sensor array of the E-nose

Sensor number	Sensor name	Responsive substance	Sensitivity levels
1	W1C	aromatic compounds	Toluene, 10 ppm
2	W5S	nitrogen oxides	NO_2, 1 ppm
3	W3C	ammonia and aromatic compounds	Benzene, 10 ppm
4	W6S	hydrogen	H_2, 100 ppb
5	W5C	alkanes, aromatic compounds	Propane, 1 ppb
6	W1S	methane, broad range.	CH_3, 100 ppm
7	W1W	sulfur compounds, pyrazine, many terpenes	H_2S, 1 ppm
8	W2S	alcohol, some aromatic compounds, broad range	CO, 100 ppm
9	W2W	aromatic components, sulphur organic compounds	H_2S, 1 ppm
10	W3S	high concentrations of methane-aliphatic compounds	CH_3, 100 ppm

Table S2 Sensory evaluation of rice porridge

		Characteristic description
Odor	Purity<br>Intensity	It has a unique aroma of rice porridge, and the aroma is strong: 18-20 points<br>It has the unique aroma of rice porridge, and the aroma is fragrant: 15-17 points<br>It has a unique aroma of rice porridge, and the aroma is not obvious: 12-14 points<br>Rice porridge has no fragrance, but no peculiar smell: 7-11 points<br>Rice porridge has a peculiar smell: 0-6 points
Appearance	Color	Uniform color, good gloss: 8-10 points<br>Uneven color, light gloss: 6-7 points<br>Darker color or obvious heterochromia: 0-5 points
	Uniformity	The turbidity of the rice soup is suitable, the viscosity is high, and a large number of rice grains are suspended:13-15 points<br>The turbidity of rice soup is suitable, the viscosity is not high, and more rice grains are suspended:10-12 points<br>Rice soup is turbid, too thick or too thin: 6-9 points<br>Rice soup is transparent, stratification serious: 0-5 points
Palatability	Stickiness	Smooth, rice grains have a certain viscosity, no bonding: 8-10 points<br>A small amount of rice grains are bonded, and the stickiness of rice grains is generally: 6-7 points<br>Rice grains bond into clusters, sticking teeth, or no stickiness: 0-5 points
	Springiness	There is some flexibility: 8-10 points

Continued Table S2 Sensory evaluation of rice porridge

Palatability	Springiness	Slightly flexible: 6-7 points
		Inelastic: 0-5 points
	Hardness	Moderate hardness: 8-10 points
		Feeling slightly hard or too soft: 6-7 points
		Soft, hard, sandwiched: 0-5 points
Taste	Purity	Rich fragrance and sweetness: 22-25 points
	Durability	Light fragrance and sweetness:18-21 points
		No fragrance and sweetness, but no peculiar smell: 16-17 points
		No fragrance and sweetness, peculiar smell: 0-15 points

Table S3 Effect of konjac glucomannan on the pasting properties of rice flour

	Peak viscosity/cP	Trough viscosity/cP	Breakdown /cP	Final viscosity /cP	Setback /cP	Pasting temperature /°C
CON	1365±4.24^{d}	756±10.61^{d}	608±14.85^{d}	1154±24.75^{d}	398±14.14^{d}	74.38±0.25^{b}
KGM-0.5	1522±38.18^{cd}	848±12.02^{c}	673±26.16^{cd}	1272±19.80^{c}	423.5±7.78^{cd}	74.68±0.04^{b}
KGM-1.0	1652±18.38^{c}	914±19.80^{c}	738±1.41^{c}	1352±25.46^{c}	438±5.66^{c}	74.88±0.11^{b}
KGM-1.5	2227±141.42^{b}	1049±60.10^{b}	1177±81.32^{b}	1532±75.95^{b}	482.5±14.85^{b}	75.36±0.55^{b}
KGM-2.0	2929.5±9.19^{a}	1246±21.90^{a}	1683±12.73^{a}	1773±26.16^{0}	526±4.24^{a}	76.63±0.60^{a}

CON: control group, KGM-0.5, KGM-1.0, KGM-1.5, and KGM-2.0 indicated that different additions of konjac glucomannan were added to rice flour. Different letters were considered to be significantly different, $p<0.05$.

Table S4 The proportion of different water state

	T_{21}/ms	T_{22}/ms	T_{23}/ms	$A_{21}/\%$	$A_{22}/\%$	$A_{23}/\%$
CON	0.98±0.10^{a}	12.25±0.27^{b}	123.45±2.73^{a}	1.94±0.06^{b}	6.12±0.11^{c}	91.93±0.15^{a}
KGM-0.5	1.12±0.13^{a}	13.35±1.11^{ab}	119.54±1.86^{ab}	2.03±0.16^{ab}	6.40±0.39^{c}	91.54±0.45^{ab}
KGM-1.0	1.07±0.16^{a}	13.70±1.18^{a}	117.42±6.83^{bc}	2.17±0.13^{a}	6.77±0.12^{b}	91.06±0.23^{bc}
KGM-1.5	1.22±0.37^{a}	13.63±0.78^{a}	114.01±2.39^{c}	2.09±0.22^{ab}	7.13±0.39^{a}	90.78±0.55^{c}
KGM-2.0	1.10±0.13^{a}	13.00±0.80^{ab}	122.66±2.59^{ab}	1.95±0.12^{b}	6.14±0.12^{c}	91.91±0.20^{a}

CON: control group, KGM-0.5, KGM-1.0, KGM-1.5, and KGM-2.0 indicated that different additions of konjac glucomannan were added to rice porridge. Different letters were considered to be significantly different, $p<0.05$.

Table S5 Effect of konjac glucomannan on volatile compounds of rice porridge

Code	Volatile compounds	MW	RI	Content (μg/kg)				
				CON	KGM-0.5	KGM-1.0	KGM-1.5	KGM-2.0
A1	2-methyl-Butanal	86	661	0.37	—	—	—	—
A2	Hexanal	100	1080	1.61	1.69	1.65	0.90	0.91
A3	Heptanal	114	901	—	0.14	—	—	—
A4	Octanal	128	1299	0.99	1.25	1.38	0.51	0.95
A5	(E)-2-Heptenal	112	1334	0.86	1.05	1.02	0.56	0.39
A6	Nonanal	142	1390	10.00	9.19	11.07	5.71	14.17
A7	(E)-2-Octenal	126	1434	0.87	0.72	0.80	0.48	0.46
A8	Benzaldehyde	106	1495	2.21	1.83	1.40	1.24	1.29
A9	Decanal	156	1486	3.73	2.43	2.38	1.80	4.39
A10	(E)-2-Nonenal	140	1542	0.35	0.38	0.27	0.26	0.52
A11	Pentadecanal	226	2054	—	—	0.26	0.17	0.52
A12	Tetradecanal	212	1588	—	—	—	—	0.22
A13	Dodecanal	184	1698	0.05	—	0.25	—	—
A14	(E,E)-2,4-Decadienal	152	1826	0.17	0.49	0.16	—	0.34

CON: control group, KGM-0.5, KGM-1.0, KGM-1.5, and KGM-2.0 indicated that different additions of konjac glucomannan were added to rice porridge.

Continued Table S5 Effect of konjac glucomannan on volatile compounds of rice porridge

Code	Volatile compounds	MW	RI	Content (μg/kg)				
				CON	KGM-0.5	KGM-1.0	KGM-1.5	KGM-2.0
B1	1-Penten-3-ol	86	1164	—	0.27	—	—	—
B2	1-Octen-3-ol	128	1456	0.26	0.40	0.49	0.26	0.19
B3	1-Pentanol	88	1274	—	—	—	0.05	—
B4	1-Heptanol	116	1465	—	—	—	—	0.05
B5	(E)-2-Hexen-1-ol	100	1410	—	—	0.14	—	—
B6	2-Nonen-1-ol	142	1051	0.32	0.17	—	0.22	0.32
B7	2-ethyl-1-Hexanol	130	1484	1.94	1.92	0.42	1.23	1.67
B8	Linalool	154	1552	—	—	—	—	1.03
B9	1-Octanol	130	1545	0.41	0.61	0.97	0.39	0.41
B10	1-Nonanol	144	1673	—	0.28	0.21	0.23	0.72
B11	(Z)-3-Nonen-1-ol	142	1682	0.10	—	—	—	—
B12	1-Dodecanol	186	1953	1.68	1.81	1.73	1.31	0.77
B13	trans-Farnesol	222	2348	—	0.68	—	—	—
C1	2-Hexanone	100	1102	—	0.04	—	—	—
C2	4-Octanone	128	1197	0.14	0.19	—	—	—
C3	6-methyl-5-Hepten-2-one	126	1336	0.33	0.46	0.46	0.29	0.47
C4	5-Nonanone	142	1325	—	—	—	—	0.12
C5	3-Nonen-2-one	140	1506	—	0.62	—	—	—
C6	(Z)-6,10-dimethyl-5,9-Undecadien-2-one	194	1835	0.75	0.85	1.09	1.04	1.60
C7	2-Pentadecanone	226	2021	—	0.62	0.34	0.29	0.29

CON: control group, KGM-0.5, KGM-1.0, KGM-1.5, and KGM-2.0 indicated that different additions of konjac glucomannan were added to rice porridge.

Continued Table S5 Effect of konjac glucomannan on volatile compounds of rice porridge

Code	Volatile compounds	MW	RI	Content (μg/kg)				
				CON	KGM-0.5	KGM-1.0	KGM-1.5	KGM-2.0
D1	Dodecane	170	1200	—	1.81	1.96	—	0.79
D2	Undecane	156	1100	0.47	—	—	—	—
D3	Tridecane	184	1300	1.22	1.91	1.59	—	—
D4	Tetradecane	198	1400	2.62	1.17	0.78	1.21	2.62
D5	Pentadecane	212	1500	1.79	—	0.60	—	1.00
D6	Nonadecane	268	1900	—	—	0.33	0.6	—
D7	Heptadecane	240	1700	—	—	—	—	1.63
D8	Hexadecane	226	1600	0.78	—	0.97	0.41	1.56
E1	Propanoic acid, butyl ester	130	880	0.85	0.95	0.33	0.49	0.13
E2	Toluene	92	1036	0.24	—	—	—	—
E3	Pyridine	79	1179	—	0.22	0.81	0.13	0.13
E4	Styrene	104	1254	—	—	—	0.32	0.08
E5	2-pentylfuran	137	1229	3.63	3.65	1.89	1.59	1.38
E6	Propanoic acid, octyl ester	186	1542	—	—	—	—	0.44
E7	Naphthalene	128	1707	0.21	—	—	—	—
E8	Butylated Hydroxytoluene	220	1902	—	1.38	0.77	0.45	0.60
E9	Propanoic acid, decyl ester	214	1783	1.07	—	1.16	—	—
E10	2-Methoxy-4-vinylphenol	150	2181	0.33	0.35	0.21	0.19	0.42
E11	Dibutyl phthalate	278	2680	1.01	0.96	2.26	2.06	2.85
E12	2,4-Di-tert-butylphenol	206	2330	0.63	1.00	2.03	1.16	1.53
E13	2,3-dihydro-Benzofuran	120	1224	3.13	2.9	2.46	1.61	0.34
E14	Butanoic acid, decyl ester	228	1807	0.20	—	—	—	—

CON: control group, KGM-0.5, KGM-1.0, KGM-1.5, and KGM-2.0 indicated that different additions of konjac glucomannan were added to rice porridge.
